# Plant Growth Promoting Rhizobacteria in Amelioration of Salinity Stress: A Systems Biology Perspective

**DOI:** 10.3389/fpls.2017.01768

**Published:** 2017-10-23

**Authors:** Gayathri Ilangumaran, Donald L. Smith

**Affiliations:** Department of Plant Science, Faculty of Agricultural and Environmental Sciences, McGill University, Sainte-Anne-de-Bellevue, QC, Canada

**Keywords:** salinity stress, plant tolerance, rhizobacteria, phytohormones, signaling

## Abstract

Salinity affects plant growth and is a major abiotic stress that limits crop productivity. It is well-understood that environmental adaptations and genetic traits regulate salinity tolerance in plants, but imparting the knowledge gained towards crop improvement remain arduous. Harnessing the potential of beneficial microorganisms present in the rhizosphere is an alternative strategy for improving plant stress tolerance. This review intends to elucidate the understanding of salinity tolerance mechanisms attributed by plant growth promoting rhizobacteria (PGPR). Recent advances in molecular studies have yielded insights into the signaling networks of plant–microbe interactions that contribute to salt tolerance. The beneficial effects of PGPR involve boosting key physiological processes, including water and nutrient uptake, photosynthesis, and source-sink relationships that promote growth and development. The regulation of osmotic balance and ion homeostasis by PGPR are conducted through modulation of phytohormone status, gene expression, protein function, and metabolite synthesis in plants. As a result, improved antioxidant activity, osmolyte accumulation, proton transport machinery, salt compartmentalization, and nutrient status reduce osmotic stress and ion toxicity. Furthermore, in addition to indole-3-acetic acid and 1-aminocyclopropane-1-carboxylic acid deaminase biosynthesis, other extracellular secretions of the rhizobacteria function as signaling molecules and elicit stress responsive pathways. Application of PGPR inoculants is a promising measure to combat salinity in agricultural fields, thereby increasing global food production.

## Introduction

Climate change has exacerbated the severity of environmental stressors and affects crop production worldwide as part of the present Anthropocene Era. At the same time, there is a need to maintain food security for a growing global population through increases in crop production, while also forging agriculture more sustainable. Going forward, the quality of land and water will be critically pivotal for agriculture. Excess salt concentration in soil and water resources declines agricultural productivity, turns fertile fields to marginal lands, and leads to their abandonment. The Food and Agriculture Organization estimates that salinity has affected more than 6% of land area. Much of this land is not under cultivation but, a substantial proportion of the cultivated land, which constitutes 45 million ha of irrigated land (20% of total) and 32 million ha under dryland agriculture (about 2% of total) has been affected (Munns and Tester, 2008). The proportion of salinized land area might increase owing to climate change conditions conducive for salt accumulation (Othman et al., 2006).

Soluble salts deteriorate the fertility of soil by causing adverse effects on plant growth and development (Munns and Tester, 2008). Osmotic stress is the immediate impact of salinity (occurs within minutes) due to hypertonic conditions and ion toxicity (occurs over several hours to days and weeks) is the result of toxic ions (Na^+^ and Cl^-^) accumulating in the cells. Perturbed water balance and ion homeostasis affect hormonal status, transpiration, photosynthesis, translocation of nutrients, and other metabolic processes (Munns, 2002a). Beneficial soil microbiota enhance soil-water-plant relations through intricate mechanisms and subtle signaling cues that are not yet well-understood. A widely-proven notion is that the ability of soil microbes to manipulate phytohormonal signaling and trigger several other mechanisms to work in an integrated fashion contribute to enhanced stress tolerance in plants (Dodd and Perez-Alfocea, 2012). Inoculation of crop plants with beneficial microbes is gaining agronomic importance since they facilitate cultivation under saline-prone conditions by improving salt tolerance and hence, restoring yield (Lugtenberg et al., 2013). Bacteria isolated from extreme environments such as deserts and oceans have been shown to induce salt tolerance in crop plants. For example, a *Pseudomonas fluorescens* strain isolated from date-palm rhizosphere in Saharan region promoted root growth in maize (*Zea mays*) seedlings under salt stress (Zerrouk et al., 2016). Wheat plants (*Triticum aestivum*) inoculated with *Serratia* sp. Sl-12, a halophilic bacterium isolated from a salt lake showed improved salt tolerance and increased shoot biomass (Singh and Jha, 2016).

This review focuses on the evaluation of plant growth promoting rhizobacteria (PGPR) within the context of systems biology approaches for the alleviation of salinity stress with a brief overview of the causes for salinity and courses of plant tolerance. Recent advances in ‘omics’ technologies deliver a holistic understanding of the regulatory networks of stress responses modulated by the PGPR. Further, the reader may refer to comprehensive reviews on utilization of other beneficial microorganisms including arbuscular mycorrhizal fungi (AMF), endosymbionts, halotolerant, and phyllosphere bacteria to alleviate salinity stress (Yang et al., 2009; Dodd and Perez-Alfocea, 2012; Glick, 2012; Vorholt, 2012; Egamberdieva and Lugtenberg, 2014).

## Salinity

Salinity is one of the major abiotic stressors that undermines plant growth and development (Pitman and Lauchli, 2002). Soil salinization is caused by natural or human activities that increase the concentration of dissolved salts, predominantly sodium chloride in the soil. Primary salinity is caused by natural processes, leading to significant salt accumulation in soil and groundwater over extended periods of time, which result in the formation of salt lakes, salt marshes, marine sediments, and salt scalds in the landscape. Sources of primary salinity may arise from weathering of rocks and minerals that releases soluble salts, precipitation that washes these salts downstream, wind-borne salts from oceans and sand dunes that are deposited inland, and influx of seawater followed by subsequent retreat (Pitman and Lauchli, 2002; Rengasamy, 2002).

Cultivation operations such as land clearing, excessive irrigation, and inadequate drainage are the reasons for secondary salinity. Native vegetation sustains the water table below the subsoil zone with deep roots in semi-arid and arid regions. Replacing perennial species with shallow rooted annual crops and long fallows increases water table leakage and groundwater recharge, which consecutively raises the water table level. Salt is deposited in the topsoil as the water evaporates, resulting in dryland salinity and may eventually form a salt scald. Salinity effects can be more detrimental when the groundwater table is high, as prominent in arid and coastal areas where only salt-tolerant plants (halophytes) grow (Doering and Sandoval, 1981; Rengasamy, 2002). Irrigated lands are more prone to salinity than drylands because irrigation water deposits salt behind, year after year. Secondary salinization has degenerated vast tracts of irrigated lands to the point that they are no longer economical for cultivation. Plants are often supplied with more water than they can utilize during evapotranspiration. For example, irrigation coupled with instances of heavy rainfall accelerates infiltration and groundwater recharge rates that raise the water table faster than it can drain. As the water table rises, it mobilizes dissolved salts from underground rocks close to the root zone. When the water table is within two meters of the soil surface in clay soils (less than a meter in sandy soils), there is a high probability of salt accumulation in the topsoil and salt stress to plants. Salt is also discharged and redistributed by surface runoff or leached down into soil profile by rainfall and then move laterally to watercourses (Sharma and Prihar, 1973; Pitman and Lauchli, 2002).

Poorly drained soils also suffer from waterlogging in irrigated areas. Clay soil (fine-textured) is less permeable than loam (medium-textured) and sandy soil (coarse-textured) and hence, it has high water holding capacity with low infiltration rate. Water can be stored and used by plants for a long time in clay soil but will not quickly transmit salt away from the root zone. The low porosity of clay soil acts as an impervious layer, causing inadequate drainage (Nassar and Horton, 1999). Inefficient irrigation and drainage systems lead to poor water distribution, resulting in over-irrigated waterlogged areas or under-irrigated water deficit areas, both causing salt accumulation. Waterlogging aggravates salinity stress by limiting aeration and nutrient supply to plants while proper grading and installation of drains to carry excess water and dissolved salts away from water stagnant areas may solve these problems. Groundwater mounds can develop in irrigated areas and force saline groundwater into waterways. Irrigation with salt-rich water increases salt being added to the soil and requires more water to leach out salts to prevent them from accumulating in the topsoil. Leaching reduces salinity levels when there is sufficient drainage and the groundwater table is deep. Conservation farming practices recommend appropriate methods to improve soil structure and irrigation efficiency (Shalhevet, 1994; Bauder and Brock, 2001).

The amount of salt stored in the soil also depends on soil type, with sandy soil having low and high capacity for clay loam minerals due to Na^+^ bound to negatively charged clay particles. Soil with ECe (electrical conductivity of saturated paste extract) of 4 dS m^-1^ is defined as saline by the USDA salinity laboratory. Most crop species are affected by ECe of less than 4 dS m^-1^ and thus, saline soil inhibits the yield of crops. Salinity caused by irrigation schemes has been recognized as a serious problem around the world since irrigated land is, on average, twice as productive as rain-fed land and produces about one-third of global food (Munns and Tester, 2008). Because salinity and water are inextricably linked, climate changes drive extreme consequences on agriculture when drought or flooding hit vulnerable regions. Salinization management has focused on improving irrigation water quality and soil drainage to strategically increase salt acclimation in crops (Pitman and Lauchli, 2002).

## Salt Tolerance in Plants

Salinity tolerance in plants is dependent on its physiological mechanisms, duration of exposure to saline conditions, concentration of salt around roots, local soil–water relations, and microclimate conditions (temperature, humidity, etc.). Salt tolerance is usually quantified over a given period as survival, vegetative growth, or harvestable biomass at different physiological stages of the plant in saline versus non-saline conditions (Munns, 2002b). Crop yield decreases when salt concentration is above the threshold salinity level due to salt affecting the development of reproductive structures or translocation of nutrient reserves. There is a great diversity in salt tolerance between species and each species has a specific threshold salinity. Environmental adaptations and inherent genetic traits regulate salinity tolerance mechanisms in glycophytes and halophytes (Munns, 2002b). The majority of the plants are glycophytes (sensitive to salt) and tend to exclude the salts from roots, delaying salinity stress (Zhu, 2007). Halophytes grow in saline conditions and therefore, possess enhanced tolerance to high salt levels. They accumulate salts, carry through the xylem stream and precipitate them on leaves. Some species have evolved specialized cells called salt glands in shoots to excrete salt on its surface, which is then removed by water or wind. Few attempts have been made to introduce halophyte genes in crop plants and cultivate halophytes for food, forage, or fuel (Flowers et al., 1986; Flowers and Colmer, 2015).

Salinity impairs plant growth by causing osmotic imbalance and ion toxicity. The first osmotic phase occurs immediately when salt concentration increases above a threshold level around the roots. The osmotic stress induces water deficit in roots and shoot growth is arrested within minutes of exposure, but then recovers over several hours to a slow steady rate of growth. The second phase develops with time and is driven by the toxicity of excess Na^+^/Cl^-^ ions that accumulate in the cytoplasm. When the salt concentration exceeds the rate of exclusion by roots or cellular ability to compartmentalize salts in the vacuoles, it builds up in the cytosol and disrupts cellular structures and functions (Munns, 2002b). Hence, all salinity tolerance in plants is directed towards maintaining osmotic balance and ion homeostasis. Even though the loss of cell turgor after the immediate osmotic shock is transient, reduction of cell elongation and cell division rates in root tips and young leaves over time lead to growth inhibition (Passioura and Munns, 2000). Osmotic stress affects shoot and reproductive development, for instance, younger leaves emerge slowly, lateral buds remain quiescent and flowering starts earlier. The growth regulating mechanisms are speculated to be long-distance signals of hormones and their precursors from roots to shoots. Phytohormone signaling is essential for regulation of cell division and differentiation, thereby controlling plant developmental morphogenesis (Santner and Estelle, 2009). The integrated signaling pathways are crucial in plant protection and adaptation mechanisms during abiotic and biotic stresses. In addition to five classical phytohormones, auxin, gibberellin, cytokinin, abscisic acid, and ethylene, other molecules including salicylic acid, jasmonic acid, nitric oxide, brassinosteroids, and strigolactones have been known to function as plant growth regulators. Phytohormone status is interdependent and both negative feedback and positive stimulation of synthesis have been reported. Many of the proteins including some transcription factors and protein kinases involved in plant hormone signaling have been elucidated. Phytohormone signaling cascades influence osmotic balance and other salt tolerance mechanisms (discussed below) and regulate plant acclimatization to salinity (reviewed in detail by Waśkiewicz et al., 2016). The plant roots encounter salinity first and root elongation rate recovers after initial exposure to salt but root architecture undergoes transition over time and high salt concentration represses formation of lateral roots. The aboveground symptoms of salinity induced osmotic stress overlap to that of drought stress, including leaf senescence and stunted growth (Munns, 2002a).

Osmotic stress affects stomatal conductance instantly due to perturbed water balance and abscisic acid (ABA) synthesis in guard cells, causing stomatal closure. Over the next several hours, transpiration rate is stabilized to a new reduced rate and ABA levels *in situ* are established (Fricke et al., 2006). Increased osmotic tolerance results in greater leaf expansion and stomatal conductance, which is beneficial only when there is sufficient soil water for transpiration losses (Munns and Tester, 2008). Photosynthesis rate decreases not only because of reduced leaf area and lesser gas exchange but also due to feedback inhibition of unused photosynthates, after exposure to salinity. The growth of sink tissues is constrained and carbohydrates accumulate in plant meristems and storage organs, which otherwise would be used in their proliferation and expansion. Modulating carbohydrate production in source leaves, phloem transport, and sink utilization downregulate the feedback photoinhibition and boost plant energy metabolism (Paul and Foyer, 2001; Perez-Alfocea et al., 2010). Reactive oxygen species (ROS) are constantly generated by cell organelles as a metabolic by-product and function as signaling molecules but their production is spiked under stressed environments. ROS including hydrogen peroxide, superoxide, and free oxygen radical are profoundly reactive with cellular components and induces programmed cell death. ROS cause chlorophyll degradation and lipid peroxidation that affects photosynthesis and membrane permeability, respectively (Apel and Hirt, 2004).

Plants have developed antioxidant mechanisms involving enzymes (superoxide dismutase, glutathione reductase, catalase, and peroxidases) and molecules (carotenoids, flavonoids, and other phenolics) that prevent tissues from oxidative damages by quenching and detoxifying ROS (Gill and Tuteja, 2010). Upregulation of antioxidant enzyme activity and metabolite synthesis is coordinated by gene networks in response to initial low levels of ROS and other signaling events (Mittler et al., 2004). Antioxidant production and osmolyte accumulation are considered as sensitive physiological markers of salt and other abiotic stresses (Munns, 2002a). A common metabolic change in response to salinity is the synthesis of low molecular weight organic compounds including polyols (sorbitol, mannitol, inositol, or glycerol), amino acids (proline or glutamate), and betaines (glycine betaine) that function as osmolytes. They are compatible solutes and accumulate in the cytosol to maintain osmotic balance both inside and outside the cell. Osmolytes also function as osmoprotectants by preventing desiccation of membranes and stabilize dehydrated enzymes rather playing role in osmoregulation. They facilitate stabilization of subcellular structures and free radical scavenging and protect plants from osmotic stress induced dehydration (Rhodes et al., 2002). Synthesis of osmolytes is an energy-demanding process yet enables the plant to recover from adverse effects of salt stress (Raven, 1985).

Effects of ionic stress are determinant under prolonged exposure to high salinity levels and predominant in salt-sensitive species. Sodium ions are toxic to many plants, so are high concentrations of chlorine, specifically those that are poor excluders of Na^+^ (ex: rice and beans) and sensitive to Cl^-^ (ex: soybean and citrus). The influx of Na^+^ from roots is deposited in the xylem, carried through the transpiration stream and accumulated in the leaf blade rather than roots. Excluding Na^+^ is a daunting task because a relatively small proportion is recirculated through phloem and most of it remains in the shoot, causing toxicity (Munns, 2002a; Tester and Davenport, 2003). Hence, active efflux of Na^+^ from cells and retrieval of Na^+^ from xylem is required throughout the plant and achieved by regulatory networks of sodium/proton antiporters and high-affinity potassium transporters (Tester and Davenport, 2003; Davenport et al., 2005). A Na^+^/H^+^ antiporter SOS1 (salt overly sensitive) localized on the plasma membrane is involved in the transport of Na^+^ out of the cell and its activity is dependent on substrate (Na^+^) concentration (Qiu et al., 2002). Excess Na^+^ ion concentration affects low-affinity potassium uptake system because of the similar chemical nature of Na^+^ and K^+^ ions thereby, inhibiting K^+^ uptake by the roots. Plants activate high-affinity K^+^ transporters (HKT) to increase the uptake of K^+^ ions over Na^+^ ions and K^+^ concentration relative to Na^+^ in cytoplasm increases salinity tolerance (Rodriguez-Navarro and Rubio, 2006). Salt accumulation in intracellular spaces restrain enzymes involved in photosynthesis and respiration and interfere with vesicular trafficking (Baral et al., 2015; Jacoby et al., 2016). Cytosolic activities are inhibited under a high Na^+^/K^+^ ratio and cells need to effectively compartmentalize sodium into vacuoles, which further improves osmotic adjustments. Intracellular compartmentation of Na^+^ is regulated by Na^+^/H^+^ antiporters and Na^+^/H^+^ exchangers (NHX) on the tonoplast, which are driven by a proton gradient (Halfter et al., 2000).

Plants with adequate calcium supply have demonstrated enhanced salt tolerance and supplemental Ca^2+^ stimulates rapid leaf elongation rate (Cramer, 1992). Calcium mediated signaling is important in maintaining Na^+^/K^+^ ratios by sustaining potassium transporters and suppressing non-selective cation channels and a rise in cytosolic Ca^2+^ levels is the first detectable response to sodium stress (Epstein, 1998). Membrane depolarization activates Ca^2+^ channels in cellular membranes that regulate Ca^2+^ oscillations in the cytosol and generate Ca^2+^ signals under salt stress. The calcium signal sensor, calcineurin B-like protein (CBL4, previously identified as SOS3) forms a complex with a CBL-interacting protein kinase (CIPK24, identified as SOS2) to phosphorylate SOS1, thus enabling its activation (Halfter et al., 2000; Zhu, 2002). Other sensor proteins are calcium dependent protein kinases (CDPKs), SOS3-like calcium binding proteins (SCaBPs), and calmodulins (CaMs) (Chinnusamy et al., 2006). Progressive accumulation of Cl^-^ is toxic to chloroplasts and mitochondria, and tolerance of high Cl^-^ concentrations requires compartmentalization and exclusion. The active influx of Cl^-^ is catalyzed by a Cl^-^/2H^+^ symporter but passive uptake also occurs under saline conditions and efflux takes place through Cl^-^ permeable channels (Yamashita et al., 1994). Transport of Cl^-^ to shoots is limited by reduced xylem loading of Cl^-^ via anion channels (downregulated by ABA) and Cl^-^ is actively retrieved from the xylem stream (Gilliham and Tester, 2005).

Biochemical analysis, gene expression and mutant studies conducted to investigate molecular functions of plants in response to salinity revealed that complex signal transduction pathways and gene regulatory networks exist to alleviate stress (Hasegawa et al., 2000). Breeding of salt-tolerant genotypes to improve crop production has been persevered by plant scientists but in spite of the advances, relatively few determinant genetic traits for salt tolerance in crop species have been identified to date (Munns and Tester, 2008). However, the acquired knowledge will lead to the development of tolerant cultivars and implementation of sustainable crop protection measures that are environmentally safe. Conventional breeding practices and genetic engineering techniques could be the most relevant but often time-consuming and cost-intensive strategies. Meanwhile, application of beneficial microbes to increase salt tolerance in plants is a feasible alternative to reclaim salinity prone lands under cultivation (Berg, 2009). A plant, together with its associated microbial community, the phytomicrobiome function as a holobiont. The physiology and metabolism of the host plant are influenced by the phytomicrobiome, facilitating its adaptation to the habitat. Members of the phytomicrobiome, which include PGPR, AMF and other facultative endosymbionts are inoculated as microbial consortia and this strategy has gained interest lately to enhance crop productivity in stressed environments (Smith et al., 2015b).

## Salt Tolerance Mediated by Plant Growth Promoting Rhizobacteria

During the past century, research has continuously demonstrated numerous beneficial associations between plants and microbes, beginning with the classic legume–rhizobia symbiosis. The plant rhizosphere is enriched with nutrient sources excreted from roots that support the higher abundance of microbial population than the surrounding bulk soil (Lugtenberg and Kamilova, 2009). Free-living beneficial bacteria dwelling in the rhizosphere that exert beneficial activities are known as plant growth promoting rhizobacteria (PGPR). Some of them are facultative endophytes that further invade intercellular spaces of host tissues and thrive as endophytes to establish a mutually beneficial association. PGPR living outside the plant cell are differently associated with plant roots and directly relate to the underlying mechanisms of plant–microbe interactions. The majority of the PGPR colonize the root surface and thrive in spaces between root hairs and rhizodermal layers whereas, some are not physically in contact with the roots (Gray and Smith, 2005). Root exudates are an integral part of rhizosphere signaling events and regulate communication in beneficial plant–microbe interactions. Phenols, flavonoids, and organic acids secreted by roots have been known to act as chemical signals for bacterial chemotaxis, secretion of exopolysaccharides, quorum sensing and biofilm formation during rhizosphere colonization (Bauer and Mathesius, 2004; Badri et al., 2009; Narula et al., 2009). Isolated from rhizosphere soils, PGPR are screened *in vitro* for plant growth promoting characteristics and tested for beneficial effects in greenhouse and field trials prior to commercialization. PGPR promote plant growth and development through diverse mechanisms such as enhanced nutrient assimilation (biofertilizers) by biological nitrogen fixation, phosphorous solubilisation or iron acquisition (Rodriguez and Fraga, 1999; Steenhoudt and Vanderleyden, 2006; Sharma et al., 2013; Jin et al., 2014; Kuan et al., 2016), control pathogens by antagonism and competition (biocontrol agents) (Compant et al., 2005; Beneduzi et al., 2012; Chowdhury et al., 2015), degrade organic pollutants and reduce metal toxicity of contaminated soils (bioremediation), and facilitate phytoremediation (Divya and Kumar, 2011; Nie et al., 2011; Janssen et al., 2015; Weyens et al., 2015).

**FIGURE 1:**
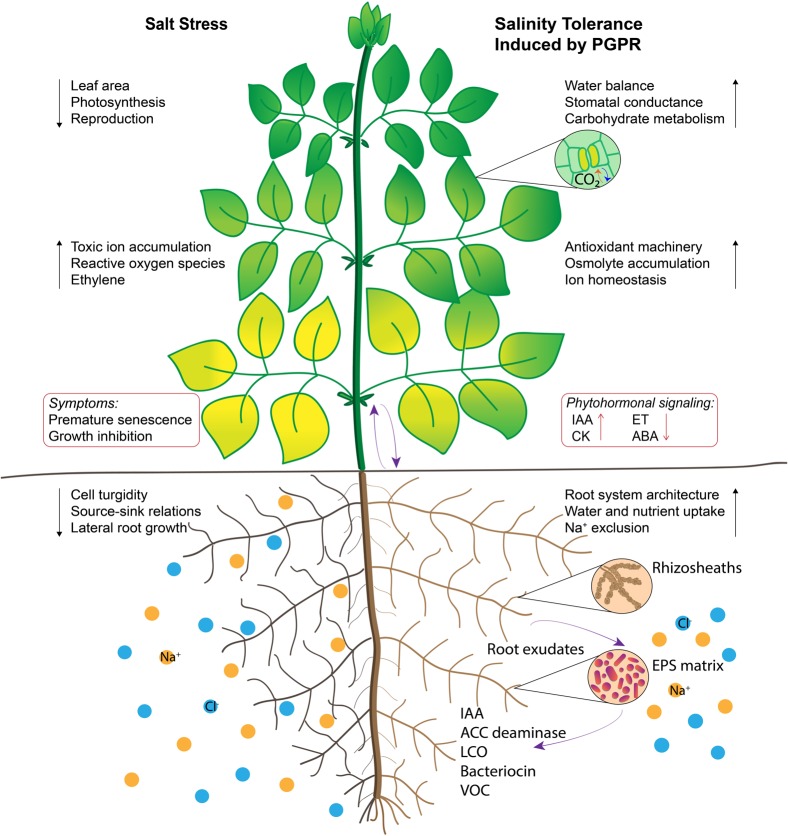
Illustration of salt tolerance mechanisms induced by plant growth promoting rhizobacteria (PGPR). Root surfaces are colonized by PGPR and extracellular polysaccharide matrix acts as a protective barrier against salt stress. Some extracellular molecules function as signaling cues that manipulate phytohormonal status in plants. Enhanced root-to-shoot communication improves water and nutritional balance, source-sink relations and stomatal conductance. Stimulating osmolyte accumulation, carbohydrate metabolism and antioxidant activity delay leaf senescence, which inturn contribute to photosynthesis. Regulation of physiological processes are indicated by black arrows and signaling pathways are indicated by purple arrows.

**FIGURE 2:**
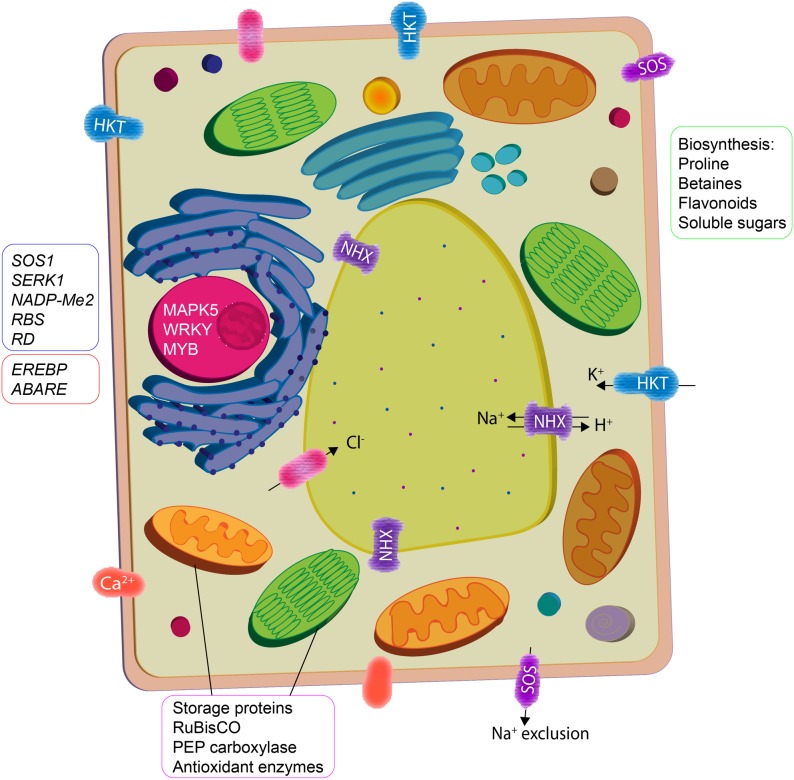
Plant growth promoting rhizobacteria interaction mediate cellular activity in plants to ameliorate salinity stress. Osmotic imbalance and oxidative damage are reduced by enhanced biosynthesis of compatible solutes and antioxidants. Ion homeostasis is maintained by increase in activity of K^+^ transporters (HKT) and H^+^ exchangers (NHX) that facilitate salt compartmentalization/exclusion. PGPR also upregulate the expression of stress responsive genes (phytohormone signaling) and proteins (vegetative storage, photosynthesis, and antioxidant enzymes).

Inoculation with PGPR has been known to modulate abiotic stress regulation via direct and indirect mechanisms that induce systemic tolerance (Yang et al., 2009). Many PGPR have been investigated for their role in improving plant-water relations, ion homeostasis and photosynthetic efficiency in plants under salt stress (**Figure 1**); their amelioration mechanisms are intricate and often not well-understood. These mechanisms are regulated by a complex network of signaling events occurring during the plant–microbe interaction and consequently ensuing stress alleviation (Smith et al., 2017). Accumulating evidence using high-throughput techniques implies that understanding the dynamic function of PGPR in relation to stomatal conductance, ion transport, water and nutrient uptake, phytohormonal status, signal transduction proteins, antioxidant enzymes, and carbohydrate metabolism in plants is important for determining the induced systemic tolerance (**Figure 2**).

### Osmotic Balance

Plant growth promoting rhizobacteria regulate water potential and stomatal opening by affecting hydraulic conductivity and transpiration rate. Maize plants inoculated with *Bacillus megaterium* showed increased root hydraulic conductivity compared to uninoculated plants when exposed to salinity (2.59 dS m^-1^) and this was correlated with increased expression of two ZmPIP (plasma membrane aquaporin protein) isoforms (Marulanda et al., 2010). PGPR induce osmolyte accumulation and phytohormone signaling that facilitate plants to overcome initial osmotic shock after salinization. Enhanced proline synthesis in transgenic *Arabidopsis thaliana* with proBA genes derived from *Bacillus subtilis* conferred salt tolerance to the plants (Chen et al., 2007). Inoculation of salt tolerant *Bacillus amyloliquefaciens* SN13 onto rice (*Oryza sativa*) plants exposed to salinity (200 mM NaCl) in hydroponic and soil conditions increased plant salt tolerance and affected expression of 14 genes, of which, four (*SOS1*, ethylene responsive element binding proteins *EREBP*, somatic embryogenesis receptor-like kinase *SERK1* and NADP-malic enzyme *NADP-Me2*) were upregulated and two [glucose insensitive growth *GIG* and (SNF1) serine-threonine protein kinase *SAPK4*] were downregulated under hydroponic conditions whereas, only *MAPK5* (Mitogen activated protein kinase 5) was upregulated under greenhouse conditions. Genes involved in osmotic and ionic stress response mechanisms were modulated by SN13 inoculation (Nautiyal et al., 2013).

Beneficial microorganisms can stimulate carbohydrate metabolism and transport, which directly implicate source-sink relations, photosynthesis, growth rate and biomass reallocation. Seed inoculated *B. aquimaris* strains increased total soluble sugars and reducing sugars in wheat under saline (ECe = 5.2 dS m^-1^) field conditions and resulted in higher shoot biomass, NPK accumulation, and Na reduction in leaves (Upadhyay and Singh, 2015). Higher plant dry matter accumulation after 36 days in pepper (*Capsicum annuum*) plants inoculated with *Azospirillum brasilense* and *Pantoea dispersa* under salinity was related to enhanced stomatal conductance and photosynthesis, but neither chlorophyll concentration nor photochemical efficiency of photosystem II was affected (del Amor and Cuadra-Crespo, 2012). Microbes exposed to osmolality fluctuations in their surrounding environment accumulate large quantities of osmoprotectants in their cytosol (Kempf and Bremer, 1998). Under such circumstances, biosynthesis of osmolytes including proline, trehalose, and glycine betaines by PGPR is most likely to be quicker than their associated host plants. The compatible solutes absorbed through plant roots aid in maintaining osmotic balance and preventing cellular oxidative damage under saline conditions. Co-inoculation of bean (*Phaseolus vulgaris*) with *Rhizobium tropici* and *Paenibacillus polymyxa* strain modified to overexpress trehalose 6-phosphate gene resulted in increased nodulation, N content and plant growth. A microarray analysis of nodules revealed upregulation of stress tolerance genes suggesting that extracellular trehalose, which functions as an osmoprotectant can induce salinity tolerance (Figueiredo et al., 2008).

### Ion Homeostasis

Bacteria limit plant salt uptake by trapping cations in the exopolysaccharide matrix, altering root structure with extensive rhizosheaths, and regulating expression of ion affinity transporters. PGPR have been known to increase the mineral nutrient exchange of both macro and micronutrients and alleviate nutrient imbalance caused by the high influx of Na^+^ and Cl^-^ ions. Microbial induced nutrient cycling (mineralization), rhizosphere pH changes (organic acids), and metal chelation (siderophores) increase plant nutrient availability (Dodd and Perez-Alfocea, 2012; Lugtenberg et al., 2013). PGPR help maintaining ion homeostasis and high K^+^/Na^+^ ratios in shoots by reducing Na^+^ and Cl^-^ accumulation in leaves, increasing Na^+^ exclusion via roots, and boosting the activity of high-affinity K^+^ transporters. Inoculation of *Azotobacter* strains C5 (auxin producing) and C9 in maize plants under salt stress improved K^+^ uptake and Na^+^ exclusion. Chlorophyll, proline and polyphenol contents in leaves increased and PGPR inoculation enhanced plant stress responses (Rojas-Tapias et al., 2012). In a study conducted with *Arabidopsis thaliana* and *Burkholderia phytofirmans* PsJN to understand the spatiotemporal regulation of short and long-term salt stress, colonized plants exhibited higher tolerance to sustained salt stress. The expressional patterns of genes involved in ion homeostasis (*KT1*, *HKT1*, *NHX2*, and *SOS1*) were altered after stress and rapid molecular changes induced by PsJN may be linked to the observed salt tolerance (Pinedo et al., 2015). A halophyte grass, *Puccinellia tenuiflora* inoculated with *B. subtilis* GB03 showed less Na^+^ accumulation and validated by upregulation of *PtHKT1* and *PtSOS1* genes but *PtHKT2* was downregulated in roots under high salt concentrations (200 mM NaCl) (Niu et al., 2016).

### Phytohormone Signaling

Soil bacteria modulate plant hormone status by releasing exogenous hormones, metabolites, and enzymes that may contribute to increased salt tolerance. Besides, phytohormones and metabolites are synthesized *de novo* in the plants in response signaling events of plant–microbe interactions during stress (Dodd et al., 2010).

#### Auxin

Auxin biosynthesis occurs via multiple pathways in rhizobacteria and one is the utilization of tryptophan present in root exudates and its conversion into indole-3-acetic acid (IAA), which is absorbed by the plant roots. Together with the plant’s endogenous IAA pool, an auxin signaling pathway is triggered and results in stimulation of cell growth and proliferation. IAA produced by PGPR is one of the most common and widely studied bacterial signaling molecules in plant–microbe interactions. The function of exogenous IAA is dependent on the endogenous IAA levels in plants. At optimal IAA concentration, acquisition of bacterial IAA may result in neutral, promotion or inhibition of plant growth (Dodd et al., 2010; Spaepen and Vanderleyden, 2011).

*Bacillus amyloliquefaciens* SQR9 enhanced salt stress tolerance (100 mM NaCl) of maize seedlings *in vitro* and bacterial inoculation increased chlorophyll and total soluble sugar contents, improved peroxidase and catalase activity, enhanced glutathione content, and K^+^/Na^+^ ratio. In addition, salinity induced ABA level was counteracted by SQR9 inoculation, which maintained it at the normal level. These physiological mechanisms to relieve salt stress were confirmed by the upregulation of genes *RBCS*, *RBCL* (encoding RuBisCo subunits), *H(+)-Ppase* (encoding H^+^ pumping pyrophosphatase), *HKT1*, *NHX1*, *NHX2* and *NHX3*, and also the downregulation of *NCED* expression (encoding 9-*cis*-epoxycarotenoid dioxygenase) in inoculated seedlings (Chen et al., 2016). *Enterobacter* sp. EJ01 isolated from a halophyte plant, sea china pink (*Dianthus japonicus thunb*) improved plant growth and salt stress tolerance (200 mM) in Arabidopsis and tomato (*Solanum lycopersicum*) plants. Short-term treatment (6 h) with EJ01 increased expression of genes involved in salt stress response such as DRE-binding proteins *DREB2b*, Relative to Desiccation (*RD29A*, *RD29B*), late embryogenesis abundant (LEA) genes (*RAB18*), proline biosynthesis (*P5CS1* and *P5CS2*), and stress-inducible priming processes (*MPK3* and *MPK6*) in Arabidopsis seedlings. GFP-tagged EJ01 displayed colonization of the bacteria in the rhizosphere and endosphere of Arabidopsis roots. In addition, ROS scavenging activities including antioxidant enzyme, ascorbate peroxidase were enhanced in inoculated tomato plants under salt stress (Kim et al., 2014).

The role of bacterial cytokinins in salt stress tolerance is largely unknown yet with relatively fewer studies. *Pseudomonas* strains (*P. aurantiaca* TSAU22, *P. extremorientalis* TSAU6 and *P. extremorientalis* TSAU20) enhanced growth up to 52%, compared to control plants and alleviated salinity (100 mM NaCl) induced dormancy of wheat seeds (Egamberdieva, 2009). Cytokinin producing *B. subtilis* inoculated onto lettuce seedlings under water deficit conditions increased accumulation of shoot biomass and shortened roots with only small effect on root biomass. Despite increased shoot cytokinins, the possible role in root-to-shoot signaling was latent seemingly hindered by shoot ABA (Arkhipova et al., 2007).

#### Ethylene

Synthesis of ethylene in response to stress may increase plant tolerance or expedite senescence (Morgan and Drew, 1997). Ethylene regulates plant adaptation to stress at the expense of growth and development. As ethylene levels increase under stress, transcription of auxin response factors is inhibited and it constraints plant growth. PGPR that secrete 1-aminocyclopropane-1-carboxylase (ACC) deaminase restrict ethylene biosynthesis in plants. The enzyme converts ACC, the precursor of ethylene to ammonia and α-ketobutyrate. Many studies have shown enhanced stress tolerance and growth promotion in plants conferred by soil bacteria producing ACC deaminase (Glick et al., 2007). The following examples illustrate some of the salt tolerance mechanisms induced by PGPR producing ACC deaminase.

*Pseudomonas putida* UW4 inoculated tomato (*Solanum lycopersicum*) seedlings showed increased shoot growth after 6 weeks in saline conditions up to 90 mM NaCl. The expression of *Toc GTPase*, a gene of the chloroplast protein import apparatus was upregulated, which may facilitate import of proteins involved as a part of stress response (Yan et al., 2014). A nutrient flow study of pea (*Pisum sativum* cv. Alderman) inoculated with *Variovorax paradoxus* 5C-2 under salt stress of 70 and 130 mM NaCl showed increased root to shoot K^+^ flow and Na^+^ deposition in roots, thereby increasing K^+^/Na^+^ ratio in shoots. Inoculation with PGPR also increased the photosynthesis rate and electron transport, while decreased stomatal resistance and xylem balancing pressure; overall improved the plant biomass (Wang et al., 2016). *Enterobacter* sp. UPMR18 inoculated okra (*Abelmoschus esculentus*) plants exhibited increase in antioxidant enzyme activities and transcription of ROS pathway genes when grown in 75 mM NaCl and showed enhanced salt tolerance (Habib et al., 2016). ACC deaminase producing strains of *Pseudomonas fluorescens* and *Enterobacter* spp. significantly improved maize yield in salt-affected fields. Higher K^+^/Na^+^ ratios and NPK uptake were also recorded in inoculated plants under salt stress (Nadeem et al., 2009).

Plant growth promoting rhizobacteria that produce both IAA and ACC deaminase can effectively protect plants from a range of stresses. IAA accumulation induces transcription of ACC synthase genes, which increases ACC concentration, leading to the production of ethylene. PGPR containing ACC deaminase may break down some of the excess ACC and lower plant ethylene levels during an advent of environmental stress and simultaneously allow IAA to promote plant growth (Glick, 2012). Endophytic bacteria (*Arthrobacter* sp. and *Bacillus* sp.) producing ACC deaminase and IAA increased proline content in sweet pepper (*Capsicum annuum*). The inoculated plants manifested downregulation of stress-inducible genes *CaACCO* (ACC oxidase) and *CaLTPI* (Lipid transfer protein) under mild osmotic stress (Sziderics et al., 2007). *Pantoea dispersa* PSB3 is a native bacterium in chickpea (*Cicer arietinum*) and produces IAA and ACC deaminase. Upon inoculation to chickpea cv. GPF2, it significantly improved plant biomass, pod number, pod weight, seed number, and seed weight in salt (150 mM NaCl) affected plants. The improved salt tolerance was associated with significant reduction of Na^+^ uptake and electrolyte leakage and increase of relative leaf water content, chlorophyll content, and K^+^ uptake (Panwar et al., 2016).

#### Abscisic Acid

There are relatively few studies on determining the role of exogenous ABA in plant–microbe interactions and whether bacterial ABA influences ABA status of plants under salt stress. However, PGPR modulate ABA biosynthesis and ABA-mediated signaling pathways that may contribute to the enhanced growth of salt-stressed plants. Halotolerant *Dietzia natronolimnaea* STR1 induced salinity (150 mM NaCl) tolerance mechanisms in wheat plants via modulation of an ABA-signaling cascade, validated by the upregulation of TaABARE (ABA-responsive gene) and TaOPR1 (12-oxophytodienoate reductase 1) leading to *TaMYB* and *TaWRKY* stimulation, followed by expression of stress response genes including upregulation of *TaST* (a salt stress-induced gene). Expression of SOS pathway related genes and tissue-specific responses of ion transporters were modulated. Gene expression of various antioxidant enzymes and proline content were increased, contributing to enhanced protection against salt stress in PGPR inoculated plants (Bharti et al., 2016). Cucumber (*Cucumis sativus*) plants inoculated with *Burkholderia cepacia* SE4, *Promicromonospora* sp. SE188 and *Acinetobacter calcoaceticus* SE370 had significantly higher biomass under salinity stress (120 mM NaCl). PGPR increased water potential and decreased electrolyte leakage. The inoculated plants showed down-regulation of ABA compared with control plants, while salicylic acid and gibberellin GA4 contents were increased (Kang et al., 2014a). Seed inoculation of cotton (*Gossypium hirsutum*) with *Pseudomonas putida* Rs-198 reduced ABA accumulation and increased plant biomass in salinized soil but the induced salt tolerance can also be attributed to regulated ionic balance and improved endogenous IAA content (Yao et al., 2010). Wheat plants inoculated with PGPR strains *Arthrobacter protophormiae* SA3 and *B. subtilis* LDR2 built up IAA while conflicted the increase of ABA and ACC content under salt stress conditions (100 mM NaCl). The amelioration effect was further validated by the upregulation of *TaCTR1* (Serine/Threonine protein kinase – ethylene responsive) and *TaDRE2* (drought-responsive element) genes (Barnawal et al., 2017).

### Extracellular Molecules

The extracellular secretions of PGPR including proteins, hormones, volatiles, polyamines, and other compounds have been determined to manipulate signaling pathways and regulatory functions that positively impact plant defense and development by stimulating growth, inducing disease resistance and eliciting stress tolerance (Barnawal et al., 2013; Kang et al., 2014b; Bhattacharyya et al., 2015; Smith et al., 2015a; Zhou et al., 2016).

#### Exopolysaccharides

Bacteria secrete exopolysaccharides (EPS) which are responsible for attachment, often along with other bacteria, to soil particles and root surfaces. EPS bind soil particles to aggregates, stabilizing soil structures, and increasing water holding capacity and cation exchange capacity (Upadhyay et al., 2011). EPS usually form an enclosed matrix of microcolonies, which confer protection against environmental fluctuations, water and nutrient retention, and epiphytic colonization (Balsanelli et al., 2014). They are also indispensable for mature biofilm formation and functional nodules in legume–rhizobia symbiosis (Stoodley et al., 2002; Skorupska et al., 2006). Inoculation of EPS producing *Pseudomonas mendocina* with an arbuscular mycorrhizal fungus, *Glomus intraradices* onto lettuce (*Lactuca sativa*) resulted in stabilization of soil aggregates under field conditions (Kohler et al., 2006). Inoculation with salt-tolerant *Halomonas variabilis* HT1 and *Planococcus rifietoensis* RT4 increased the growth of chickpea (*Cicer arietinum* var. CM-98) and soil aggregation with roots under high salt concentrations (up to 200 mM NaCl) (Qurashi and Sabri, 2012). Quinoa (*Chenopodium quinoa*) seeds inoculated with *Enterobacter* sp. MN17 and *Bacillus* sp. MN54 improved plant-water relations under saline irrigation conditions of 400 mM NaCl (Yang et al., 2016). EPS production and composition improve bacterial resistance to abiotic stress (Sandhya and Ali, 2015) but the role of EPS in plant salinity tolerance deserves further investigation.

#### Lipo-chitooligosaccharides

Legume–rhizobia symbiosis is affected by salt stress and high levels of salinity inhibit nodule formation and nitrogen fixation (Tu, 1981; Zahran, 1999). Lipo-chitooligosaccharides (LCOs) are secreted by rhizobia as Nod-factors (NFs) in response to flavonoids present in root exudates and initiate nodule formation. LCOs are conserved at the core but diverge in the *N*-Acetyl chain length, degree of saturation, and substitutions (glycosylation or sulfation), which are crucial in host specificity (Oldroyd, 2013). Nod-factors also act as stress response signals in legumes and NF synthesis is modulated by other PGPR and abiotic stresses. High salinity (100–200 mM NaCl) inhibited root hair deformation responses to increase in NF concentrations in Soybean (*Glycine max*) – *Bradyrhizobium japonicum* symbiosis (Duzan et al., 2004). Inoculation of IAA producing *Azospirillum brasilense* Cd into the *Rhizobium*-Bean (*Phaseolus vulgaris* cv. Negro Jamapa) symbiosis increased root branching and flavonoid synthesis under 50 mM NaCl. The co-inoculation also promoted *Nod*-genes expression in *R. tropici* CIAT899 and *R. etli* ISP42 grown in the presence of root exudates (Dardanelli et al., 2008). Free-living rhizobia are more resistant to salt stress than inside their legume hosts. *R. tropici* CIAT899 is highly tolerant to stress and high salt concentrations enhance *Nod*-gene expression, Nod-factor synthesis and diversity; 46 different NFs were identified compared to 29 NFs under control with only 15 NFs common to both (Estevez et al., 2009). Inoculation of *B. japonicum* 532C grown in genistein (a flavonoid) induced media significantly enhanced nodulation and growth of soybean under salinity levels (36 and 61 mM NaCl) and such positive effects become more evident with time (Miransari and Smith, 2009) and increased yield up to 21% under salinized field conditions in an earlier study.

#### Bacteriocins

Bacteriocins are small peptides secreted by rhizobacteria that are bactericidal or bacteriostatic against relative bacteria, thus providing a competitive advantage to the producer strain but might also promote microbial diversity in an ecologic niche (Kirkup and Riley, 2004). Application of thuricin 17, isolated from a soybean endosymbiont *Bacillus thuriengenesis* NEB 17 differentially altered the proteome of salt-stressed (250 mM NaCl) Arabidopsis plants. Expression of proteins involved in carbon and energy metabolism pathways were modulated by the bacterial signals. Proteins involved in photosynthesis including PEP carboxylase, RuBisCo-oxygenase large subunit, pyruvate kinase and proteins of photosystems I and II were upregulated along with other stress related proteins (Subramanian et al., 2016b). These bacterial signal compounds also induced similar changes in the proteome of soybean seeds at 48 h under 100 mM NaCl. In addition, isocitrate lyase and antioxidant glutathione-*S*-transferase were increased. These findings by shotgun proteomics suggested that thuricin 17 positively manipulate plant proteome profile and enhance physiological tolerance to salinity (Subramanian et al., 2016a).

#### Polyamines

Polyamines (PAs) are low molecular weight aliphatic amines with pronounced antioxidant activity that are ubiquitous in all living organisms and modulate ROS homeostasis by scavenging free radicals and stimulating antioxidant enzymes. The most abundant polyamines, spermidine, spermine, and putrescine are implicated in various developmental processes and stress responses in plants (Gupta et al., 2013). Application of exogenous polyamines increase abiotic stress tolerance but PGPR secretion of polyamines is largely unexplored. Spermidine from *Bacillus megaterium* BOFC15 increased cellular polyamine accumulation in Arabidopsis, thereby activating PA-mediated signaling pathways contributing to the osmotic stress tolerance of plants. The bacterial inoculation resulted in greater biomass, elevated photosynthetic capacity and higher antioxidant enzyme activity. Other tolerance mechanisms involved robust root system architecture and ABA dependent stress responses, which maintained water balance and stomatal conductance (Zhou et al., 2016).

#### Volatile Compounds

Volatile organic compounds (VOC) released from PGPR are known to stimulate plant growth, resulting in increased shoot biomass, and modulated stress responses. Perception of volatiles by plants and subsequently induced mechanisms require further research (Bailly and Weisskopf, 2012). *B. subtilis* GB03 VOCs mediated tissue specific regulations of Na^+^ homeostasis in salt-stressed plants. Arabidopsis under 100 mM NaCl treated with VOCs decreased Na^+^ accumulation by concurrently downregulating expression of *HKT1* in roots but upregulating it in shoots. Presumably, the induction of *HKT1* dependent shoot-to-root recirculation resulted in reduced Na^+^ accumulation up to ∼50% throughout the plant. Treatment with VOCs increased leaf surface area, root mass, and total K^+^ content when compared with controls whereas, inoculated *athkt1* mutants showed stunted growth. Exposure to VOCs reduced the total Na^+^ level by 18% and enhanced shoot and root growth of *sos3* mutants in 30 mM NaCl (Zhang et al., 2008). A putative VOCs blend released from *Pseudomonas simiae* AU induced salt-tolerance in soybean (*Glycine max*) under 100 mM NaCl by decreasing root Na^+^ accumulation and increasing proline and chlorophyll content. Protein expression analysis confirmed upregulation of vegetative storage proteins (Na^+^ homeostasis), RuBisCO large chain proteins (photosynthesis) in exposed soybean seedlings (Vaishnav et al., 2015).

*Paraburkholderia phytofirmans* PsJN VOCs stimulate plant growth and induce salinity tolerance that have been demonstrated both *in vitro* (150 mM NaCl/15 mM CaCl_2_) and in soil (200 mM NaCl/20 mM CaCl_2_). Growth parameters of Arabidopsis plants measured as rosette area, fresh weight, and primary root length were higher than the control plants and exposure to VOCs showed parallel growth promoting effects of direct bacterial inoculation. The emitted compounds were analyzed and the plants were exposed to a blend of 2-undecanone, 7-hexanol, 3-methylbutanol molecules, which mimicked the effect of VOCs (Ledger et al., 2016). Genome wide mapping association of Arabidopsis accession lines revealed 10 genetic loci associated with growth stimulation in response to the presence of *P. simiae* WCS417r *in vitro*, which is partly caused by VOC produced by the bacterium. Even though the study was conducted to select lines for breeding strategies, it is interesting to note that the genotype variation of host plants has different interactions with the associated root microbiome (Wintermans et al., 2016).

## Conclusion

Application of PGPR inoculants as biofertilizers and biocontrol agents is an integral component in organic farming practices (Babalola, 2010). With rising emphasis on sustainable agriculture, environmental protection, and food security, exploitation of beneficial soil microbiota is imperative. Abiotic stresses constraint yield and turn agriculture production systems fragile; in addition, persisting climate change intensify the frequency, degree, and resultant damage of stressful conditions. Plants have evolved complex mechanisms to tolerate abiotic stresses caused by various environmental factors, including salinity. Plant associated bacteria in soil mitigate the adverse effects of these stresses in a more time-sensitive and cost-effective manner, where the development of tolerant cultivars has been somewhat overwhelmed. Research directed towards the application of PGPR in salt-affected fields encourages commercialization of inoculants for salinity tolerance. The systems biology of plant–microbe interactions in response to environmental stimuli such as salinity, opens up new prospects of understanding the regulatory networks of plant salt tolerance modulated by rhizosphere bacteria (**Table 1**). While the induced salt tolerance may be contributed by the release of extracellular compounds that function as chemical signals to the plant, improved soil properties that reduce the impact of salinity is another important benefit yet to be explored. Stress adaptation of plants are induced by associated microbiota and cutting-edge research as discussed above may be successfully applied to improve crop yield in saline prone regions. The potential application of PGPR to help plants deal with stress in agricultural fields seems vastly large, yet much is left to be utilized.

**Table 1:** Summary of PGPR interaction effects in crop plants under salinity stress from recent studies using systems biology approaches.

	PGPR	Crop species	Beneficial effects	Reference
1	*Bacillus amyloliquefaciens* SN13	*Oryza sativa*	Upregulation of *SOS1*, *EREBP*, *SERK1*, *NADP-Me2*	Nautiyal et al., 2013
2	*Bacillus amyloliquefaciens* SQR9	*Zea mays*	Upregulation of *RBCS*, *RBCL, HKT1*, *NHX1*, *NHX2*, and *NHX3*	Chen et al., 2016
3	*Bacillus megaterium*	*Zea mays*	Improved expression of two ZmPIP isoforms	Marulanda et al., 2010
4	*Bacillus thuriengenesis* NEB17	*Glycine max*	Upregulation of PEP carboxylase, RuBisCo-oxygenase large subunit, pyruvate kinase, and proteins of photosystems I and II, isocitrate lyase and antioxidant glutathione-*S*-transferase	Subramanian et al., 2016a
5	*Dietzia natronolimnaea*	*Triticum aestivum*	Modulation of ABA signaling cascade, SOS pathway related genes, tissue-specific responses of ion transporters	Bharti et al., 2016
6	*Enterobacter* sp. UPMR18 (ACC deaminase)	*Abelmoschus esculentus*	Increase antioxidant enzyme activities and upregulation of ROS pathway genes	Habib et al., 2016
7	*Pseudomonas putida* UW4 (ACC deaminase)	*Solanum lycopersicum*	Increased shoot growth and expression of *Toc GTPase*	Yan et al., 2014
8	*Pseudomonas simiae* AU	*Glycine max*	Upregulation of vegetative storage proteins, RuBisCO large chain proteins. Decrease in root Na^+^ accumulation and increase in proline and chlorophyll content	Vaishnav et al., 2015


## Author Contributions

GI gathered literature and prepared the manuscript. DS provided feedback and oversaw progression of the manuscript.

## Conflict of Interest Statement

The authors declare that the research was conducted in the absence of any commercial or financial relationships that could be construed as a potential conflict of interest.
